# Impact of muscle echo intensity on post‐exercise blood pressure response in older normotensive and hypertensive females: Pilot study

**DOI:** 10.14814/phy2.15514

**Published:** 2022-11-10

**Authors:** Ryosuke Takeda, Tetsuya Hirono, Akito Yoshiko, Shun Kunugi, Masamichi Okudaira, Saeko Ueda, Kohei Watanabe

**Affiliations:** ^1^ Laboratory of Neuromuscular Biomechanics, School of Health and Sport Science Chukyo University Toyota Japan; ^2^ Research Fellow of Japan Society for the Promotion of Science Tokyo Japan; ^3^ Faculty of Liberal Arts and Sciences Chukyo University Nagoya Japan; ^4^ Center for General Education Aichi Institute of Technology Toyota Japan; ^5^ Department of Human Nutrition, School of Life Studies Sugiyama Jogakuen University Nagoya Japan

**Keywords:** intramuscular fat, muscle echo intensity, older female, post‐exercise blood pressure

## Abstract

Exaggerated post‐exercise blood pressure (BP) is considered a risk factor for the development of cardiovascular disease in older females. Muscle echo intensity (EI) using ultrasound can be used to evaluate intramuscular fat, one of the risk factors for cardiovascular disease. This study aimed to determine whether intramuscular fat assessed by muscle echo intensity is associated with the post‐exercise BP response in older females. Ten older normotensive (SBP <130 mmHg, 71 ± 4 years), eight systolic BP‐controlled (78 ± 4 years), and 17 hypertensive (SBP ≥130 mmHg, 74 ± 6 years) females were studied. After obtaining ultrasound images to assess the EI, participants performed ramp‐up exercise until 50% maximal voluntary contraction (MVC: ~30‐s; 3% MVC/s gradually increased knee extension force from 0% to 50% MVC followed by sustaining the force at 50% MVC for 10‐s) and then five MVCs (~50 s; 10‐s rest between each contraction). BP was measured before and immediately after exercise. Mean arterial pressure (MAP) pre‐ and post‐exercise were significantly lower in normotensive and SBP‐controlled, than in ‐uncontrolled hypertensive females (PRE: 85 ± 5 and 87 ± 7 vs. 106 ± 9; POST: 92 ± 8 and 94 ± 9 vs. 103 ± 11 mmHg, respectively, *p* < 0.05). EI was negatively correlated with ∆diastolic BP (∆DBP) but not ∆SBP and ∆MAP in normotensive females only (∆SBP, *r* = −0.21, *p* = 0.56; ∆DBP, *R* = −0.73, *p* = 0.02; ∆MAP, *R* = −0.49, *p* = 0.15). Greater intramuscular fat as indicated by higher EI is associated with less BP elevation immediately after exercise in older normotensive females. Greater intramuscular fat may lead to lower intramuscular pressure, resulting in less post‐exercise BP elevation.

## Graphical abstract

Muscle echo intensity was negatively correlated with changes in diastolic blood pressure (∆DBP) but not ∆systolic BP (SBP) and ∆mean arterial pressure (MAP) in only normotensive females (SBP, r=‐0.21, P=0.56; DBP, R=‐0.73, P=0.02; MAP, R=‐0.49, P=0.15).

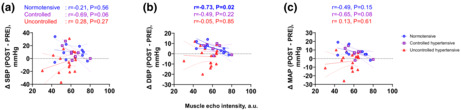

## 1 INTRODUCTION

Exaggerated post‐exercise blood pressure (BP) is associated with cardiovascular disease risk factors, such as higher arterial stiffness (Costa et al., 2022) and reduced arterial compliance (Costa et al., 2020) in older hypertensive and normotensive females. On the other hand, post‐exercise hypotension after resistance exercise also occurs in older females as well as males (Millar et al., 2009). There is a lack of consensus and understanding of the mechanisms of the post‐exercise BP response in older females. Interestingly, a previous study (Floras & Senn, 1991) reported that BP was decreased after exercise in borderline hypertensive but not in normotensive individuals. Thus, hypertension may be associated with the post‐exercise BP response in older females.

Recently, Lee et al. and Notay et al. reported that the maximal isometric strength during maximal voluntary contraction (MVC) contributes to BP responses during exercise (Lee, Lutz, et al., 2021; Lee, Notay, et al., 2021; Notay et al., 2018) and post‐exercise with circulatory occlusion in young normotensive females as well as males (Lee, Notay, et al., 2021). However, whether this is also true in older females remains unclear. Further, the addition of other populations, such as patients with hypertension, is warranted to better understand individual differences in the post‐exercise BP response in older females.

The muscle quality (i.e., ratio of skeletal muscle) is one of the contributors to maximal isometric strength during MVC in older healthy females (Fukumoto et al., 2012). To evaluate the muscle quality, measurement of muscle echo intensity using ultrasound is a non‐invasive, practical, and reproducible assessment method. Muscle echo intensity can evaluate one of the non‐skeletal muscle components ‐ intramuscular fat (Young et al., 2015). Numerous reports have stated that intramuscular fat increases with aging (Pinel et al., 2021) and excessive intramuscular fat is associated with attenuated muscle strength (Pinel et al., 2021), increases in metabolic risk factors and the incidence of hypertension (Therkelsen et al., 2013), and cardiac events (Yoshida et al., 2022). However, because greater intramuscular fat is also associated with less muscle stiffness (Pinel et al., 2021), and less muscle stiffness is associated with less intramuscular pressure (Sadeghi et al., 2019), a reasonable amount of intramuscular fat may reduce the exercise‐induced increase in intramuscular pressure (Gallagher et al., 2001). Reduced intramuscular pressure may in turn, prevent the occlusion of peripheral intramuscular circulation which is partly due to the developed intramuscular pressure. In this regard, reduced intramuscular pressure around the veins in the deeper parts of the muscle can be considered of particular importance (Sadamoto et al., 1983) and might prevent the increased post‐exercise BP response in older females.

Thus, the purpose of this study was to determine the impact of intramuscular fat on the post‐exercise BP response in older females. We hypothesized that individual differences in intramuscular fat are associated with individual variability of post‐exercise BP in older females. To test this hypothesis, we used muscle echo intensity assessed by ultrasound as an index of the intramuscular fat content in older females. Participants were divided into a normotensive group, a systolic BP well‐controlled hypertensive (SBP <130 mmHg) group and uncontrolled hypertensive (SBP ≥130 mmHg) group (Bakris et al., 2019) to clarify the impact of hypertension on post‐exercise BP in older females.

## 2 METHODS

### 2.1 Participants

Participants were recruited at a health promotion class held at the Chukyo University. Thirty‐five older females [means ± standard deviation: age: 74 ± 6 years, height: 151.9 ± 5.2 cm, body weight: 51.7 ± 6.6 kg] were divided into normotensive and well‐controlled SBP hypertensive groups (*n* = 10 and 8, SBP <130 mmHg, respectively) and an uncontrolled hypertensive group (*n* = 17, SBP ≥130 mmHg) based on the screening and first BP measurement on the testing day. They were all non‐smoker. Exclusion criteria for this study included a history of cardiovascular, metabolic, or neuromuscular disease. All participants provided written informed consent, the research ethics committee of Chukyo University approved the study protocol (approved number: 2021–13), and it was conducted in accordance with the Declaration of Helsinki.

### 2.2 Experimental protocol

**FIGURE 1:**
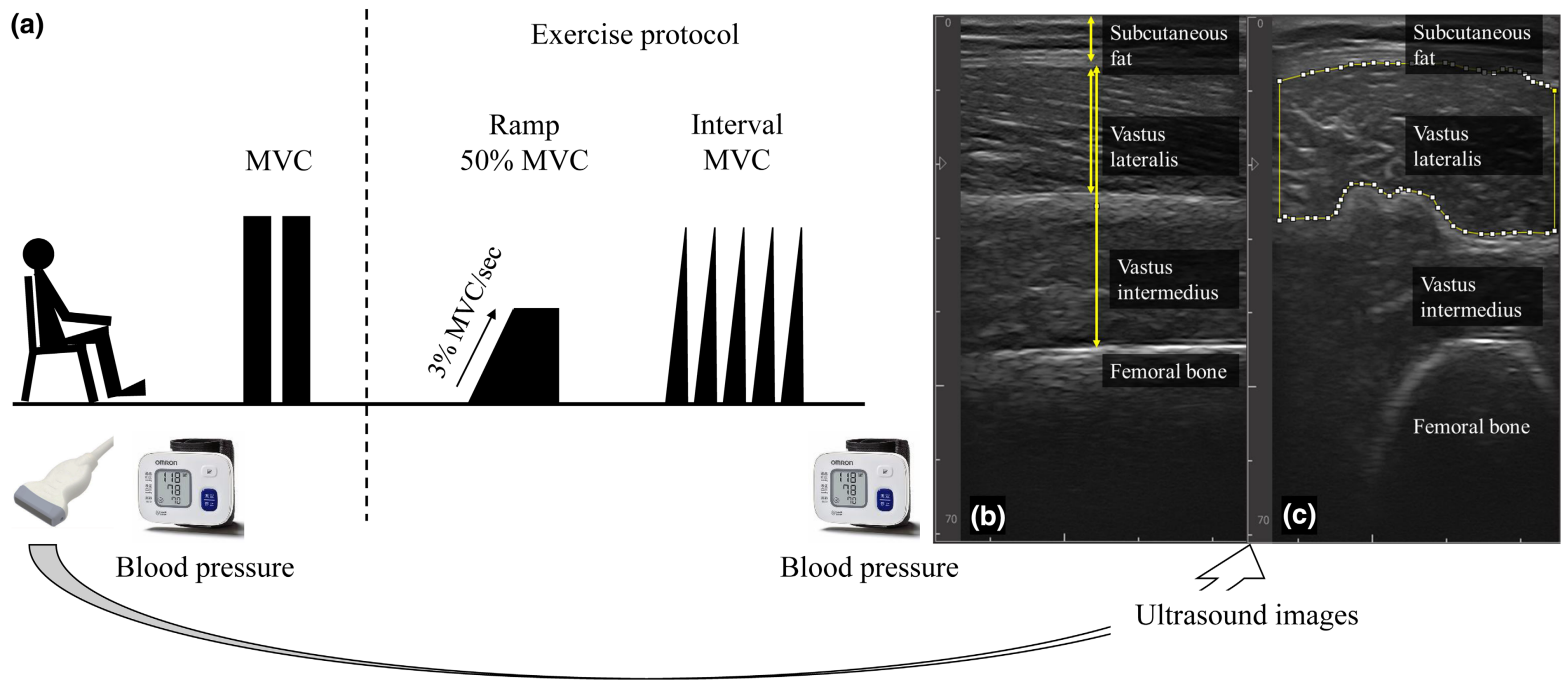
Experimental protocol (a) and ultrasound image to assess the muscle thickness (b) and muscle echo intensity (c). After ultrasound measurement, all participants underwent resting BP measurement. Then, they performed voluntary muscle contractions twice. The exercise protocol was the combination of 50% maximal voluntary contraction (MVC) in a static state (~30 s) and five MVCs (10‐s rest between each contraction). Immediately after the exercise protocol, all participants underwent post‐exercise BP measurement.

**FIGURE 2:**
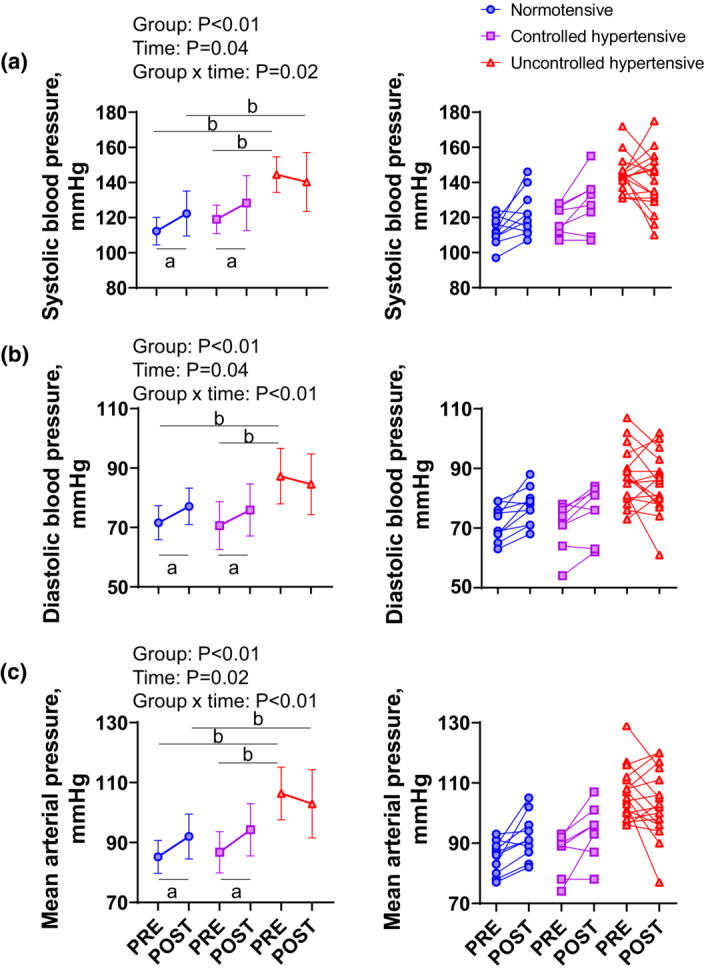
Systolic blood pressure (SBP, a), diastolic blood pressure (DBP, b), and mean arterial pressure (MAP, c) pre‐ and post‐exercise. Circles denote the normotensive females (SBP <130 mmHg; *n* = 10), squares denote the SBP‐controlled hypertensive females (*n* = 8) and triangles denote the SBP‐uncontrolled hypertensive females (SBP ≥130 mmHg; *n* = 17). Values are means ± standard deviation. Two‐way ANOVAs were used to compare the groups. ^a^Significantly different between PRE and POST, ^b^significantly different between groups, *p* < 0.05.

Figure 1a shows the experimental protocol. First, participants provided two longitudinal images to assess the thickness of the vastus lateralis (VL) muscles (Figure 1b) and 2 transversal images to assess the muscle echo intensity of VL (Figure 1c) using ultrasound after height, weight, and body mass index (BMI) measurements. After least 10 min sitting at rest, all participants underwent resting BP measurement from the wrist and performed knee extension at MVC twice. Then, participants were given an explanation about the procedure of the exercise protocol and practiced a number of times. The exercise protocol was conducted as ramp‐up exercise until 50% maximal voluntary contraction (MVC: ~30‐s; 3% MVC/s with gradually increased knee extension force from 0% to 50% MVC followed by sustained force at 50% MVC for 10‐s) and five MVCs (10‐s rest between each contraction), as part of health promotion class held at the Chukyo University. The exercise protocol was performed using the dominant leg. Immediately after the exercise protocol, all participants underwent post‐exercise BP measurement.

### 2.3 Measurements

#### 2.3.1 Ultrasound images

The participants sat on a custom‐made chair and their knee was fixed at 90‐degree flexion in a resting position. Ultrasound images of VL muscle were obtained using a B‐mode ultrasound device (MicrUS EXT‐1H, TELEMED, Atlanta, GA, USA) with a multifrequency linear array probe (L12‐5L40S‐3). The equipment settings were as follows: a frequency of 7.5 MHz, dynamic range of 66 dB, power of −7 dB, gain of 72%, and depth of 70 mm. The measurement location was determined at 50% of the distance from the greater trochanter to the upper lateral edge of the patella. An adequate coupling gel was applied to compensate for depression of the tissues.

Two longitudinal images (Figure 1b) were acquired while paying attention to placing the probe with minimal pressure and adjusting its angle so that the bone echo was brightest and fascia‐ and bone‐muscle boundaries were parallel. Muscle thickness of VL was measured as the distance between the superficial and deep fascia of VL in longitudinal images. Muscle thickness of VL was also measured as the distance between the superficial and bone‐muscle boundaries. Muscle thicknesses were determined as the mean value between two longitudinal images.

Two transversal images (Figure 1c) were also acquired while paying attention to placing the probe with minimal pressure and adjusting its angle when the bone echo was brightest. The transversal images were loaded into software (Image J version 1.53 k; National Institutes of Health, Bethesda, MD, USA), where echo intensity of VL was measured. The region of interest was set as wide as possible, excluding the surrounding fascia and bone. Echo intensity was evaluated by the average grayscale value of the region of interest, which was calculated by the standard histogram function. The grayscale values range from 0 (black) to 255 (white), with a higher value indicating a greater amount of fat and fibrous tissue within the muscle (Pillen et al., 2009). The echo intensity of VL was determined as the mean value between two transversal images.

#### 2.3.2 Blood pressure

BP was obtained from the wrist (HEM‐6161, OMRON, Kyoto, Japan) before measuring MVC and immediately after the exercise protocol. Participants were asked to raise their wrist to their heart level and keep quiet during the BP measurement. The mean arterial pressure (MAP) was calculated as diastolic BP (DBP) + (SBP‐DBP)/3.

#### 2.3.3 Maximum isometric voluntary contraction

The participants were seated in a custom‐made dynamometer (Takei Scientific Instruments Co., Ltd., Niigata, Japan) fixed to a force transducer (LU‐100KSE; Kyowa Electronic Instruments, Tokyo, Japan). The hip and knee were flexed at 90 degrees. They performed maximum voluntary isometric contraction involving knee extension twice. The peak force during the contraction was recorded and the greater value of the two measurements was taken as the MVC force. The MVC torque was calculated by multiplying the MVC force and arm length, determined as the distance between the knee joint axis and force transducer.

#### 2.3.4 Statistical analyses

Values are expressed as means ± standard deviation. Two‐way analysis of variance (ANOVA) was used to evaluate the pre‐ and post‐exercise BP between the normotensive, SBP‐controlled and ‐uncontrolled hypertensive females (Group × Time). one‐way ANOVA was used to evaluate all other variables between groups. Bonferroni corrected post hoc procedures were used when applicable. Pearson product–moment correlation coefficient was used to evaluate the correlation between MVC, muscle thickness, and muscle echo intensity and changes in SBP, DBP, and MAP from pre‐ to post‐exercise. The partial correlation coefficient adjusted by age and BMI was used when there was significant association between changes in BP values from pre‐ to post‐exercise with MVC, muscle thickness or muscle echo intensity. For the purpose of exploratory analysis, the impacts of muscle echo intensity on BP values and other variables in normotensive females were evaluated using an unpaired Student's *t*‐test. A *p*‐value of <0.05 was considered significant. Statistical analyses were performed using IBM SPSS v. 25.

## 3 RESULTS

### 3.1 Participant characteristics

Table 1 shows participant characteristics. SBP, DBP, and MAP were significantly lower in normotensive and SBP‐controlled, than ‐uncontrolled hypertensive females (all, *p* < 0.001). There were no significant differences in other variables.

**TABLE 1:** Participant characteristics

	Normotensive	Controlled hypertensive	Uncontrolled hypertensive
	SBP <130 mmHg		SBP ≥130 mmHg
Number of participants	10	8	17
Age, years	71 (4)	78 (4)^a^	74 (6)
Height, cm	153 (4)	153 (2)	151 (7)
Weight, kg	48.1 (4.2)	54.9 (5.9)	52.2 (7.3)
Body mass index, kg/m^2^	20.6 (1.6)	23.4 (2.3)	22.9 (3.0)
Systolic blood pressure, mmHg	112 (8)	119 (8)	145 (10)^a^ ^,^ ^b^
Diastolic blood pressure, mmHg	72 (6)	71 (8)	87 (9)^a^ ^,^ ^b^
Mean arterial pressure, mmHg	85 (5)	87 (7)	106 (9)^a^ ^,^ ^b^
MVC, Nm	84.9 (19.0)	66.0 (14.1)	76.2 (23.2)
Muscle thickness, mm	19 (5)	18 (9)	20 (5)
Muscle echo intensity, a.u.	61 (13)	62 (9)	56 (10)

*Note*: Means (standard deviation).

One‐way ANOVA was used to compare the groups.

Abbreviations: a.u., arbitrary units; MVC, maximal voluntary contraction.

^a^ Indicates the significant difference versus Normotensive, *p*‐value <0.05.

^b^ Indicates the significant difference versus Controlled hypertensive, *p*‐value <0.05.

### 3.2 Blood pressure response pre‐ and post‐exercise

SBP, DBP, and MAP from pre‐ to post‐exercise were significantly increased in normotensive and SBP‐controlled, but not in ‐uncontrolled hypertensive females (Figure 2a–c). Post‐exercise SBP and MAP but not DBP were still significantly lower in normotensive, but not in SBP‐controlled compared to ‐uncontrolled hypertensive females (normotensive vs. controlled hypertensive vs. uncontrolled hypertensive females: SBP, 122 ± 13 vs. 128 ± 16 vs. 140 ± 17 mmHg; DBP, 77 ± 6 vs. 76 ± 9 vs. 85 ± 10 mmHg; MAP, 92 ± 8 vs. 94 ± 9 vs. 103 ± 11 mmHg).

### 3.3 Association between change in BP response to exercise and MVC, or muscle components

**FIGURE 3:**
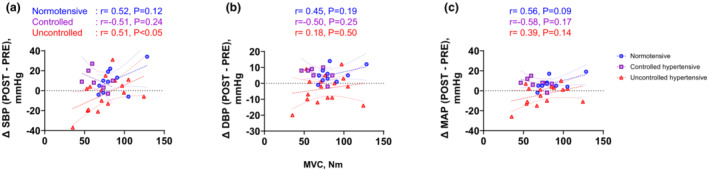
Association between maximal voluntary contraction (MVC) and changes in systolic blood pressure (∆SBP, a), diastolic blood pressure (∆DBP, b), and mean arterial pressure (∆MAP, c) from pre‐ to post‐exercise. Circles denote the normotensive females (SBP < 30 mmHg; *n* = 10), squares denote the SBP‐controlled hypertensive females (*n* = 8) and triangles denote the SBP‐uncontrolled hypertensive females (SBP ≥130 mmHg; *n* = 17). Pearson's correlation was used to assess the association.

**FIGURE 4:**
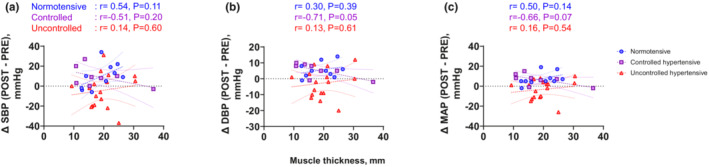
Association between muscle thickness of the vastus lateralis and changes in systolic blood pressure (∆SBP, a), diastolic blood pressure (∆DBP, b), and mean arterial pressure (∆MAP, c) from pre‐ to post‐exercise. Circles denote the normotensive females (SBP <130 mmHg; *n* = 10), squares denote the SBP‐controlled hypertensive females (*n* = 8) and triangles denote the SBP‐uncontrolled hypertensive females (SBP ≥130 mmHg; *n* = 17). Pearson's correlation was used to assess the association.

**FIGURE 5:**
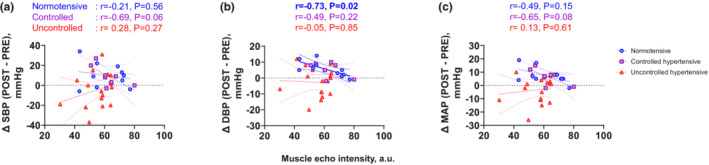
Association between muscle echo intensity of the vastus lateralis and changes in systolic blood pressure (∆SBP, a), diastolic blood pressure (∆DBP, b) and mean arterial pressure (∆MAP, c) from pre‐ to post‐exercise. Circles denote the normotensive females (SBP <130 mmHg; *n* = 10), squares denote the SBP‐controlled hypertensive females (*n* = 8) and triangles denote the SBP‐uncontrolled hypertensive females (SBP ≥130 mmHg; *n* = 17). Pearson's correlation was used to assess the association.

Absolute MVC was not associated with change in (Δ)SBP, DBP, or MAP from pre‐ to post‐exercise in any group except for ΔSBP in the SBP‐uncontrolled hypertensive group (Figure 3a–c. ΔSBP in ‐uncontrolled hypertensives, *r* = 0.51, *p* < 0.05). This relationship between MVC and ΔSBP in the ‐uncontrolled hypertensive group disappeared after adjusting for age and BMI (*r* = 0.45, *p* = 0.11). Muscle thickness of VL was not associated with ΔSBP, DBP, or MAP from pre‐ to post‐exercise in any group (Figure 4a–c). Muscle echo intensity was not associated with ΔSBP or ΔMAP from pre‐ to post‐exercise in any group (Figure 5a,c), but was negatively correlated with ∆DBP from pre‐ to post‐exercise in the normotensive, but not in the SBP‐controlled or ‐uncontrolled hypertensive groups (Figure 5b). This correlation in normotensives was still significant even after adjusted for age and BMI (*r* = −0.88, *p* < 0.01).

### 3.4 Exploratory analysis of the impact of muscle echo intensity on post‐exercise BP response and other variables in normotensive females

To perform exploratory evaluation of the impacts of muscle echo intensity on the post‐exercise BP response in normotensive females, the mean of muscle echo intensity (61 a.u.) was set as a threshold and was used to divide normotensive individuals into two subgroups (Table 2): those with muscle echo intensity >61 a.u. (*n* = 5) and <61 a.u. (*n* = 5). Table 2 showed that the increase in DBP from pre‐ to post‐exercise was significantly lower in older normotensive females with greater muscle echo intensity than in those with lesser echo intensity (muscle echo intensity, 73 ± 3 a.u. vs. 50 ± 6 a.u.; ∆DBP, 2 ± 2 mmHg vs. 9 ± 4 mmHg, *p* < 0.01 and *p* = 0.01, respectively). Other variables were not significant between groups. These results suggest that normotensive females with a higher muscle echo intensity (larger amount of intramuscular fat at VL) showed less of an increase in DBP from pre‐ to post‐exercise.

**TABLE 2:** Exploratory analysis of the impacts of muscle echo intensity on post‐exercise BP response and other variables in normotensive females with greater or lesser muscle echo intensity

	Muscle echo intensity		*p*‐value
	<61 a.u. (*n* = 5)	>61 a.u. (*n* = 5)	
Participants characteristics in normotensive females			
Age, years	70 (3)	72 (5)	0.38
Height, cm	154 (4)	152 (4)	0.49
Weight, kg	46.7 (4.1)	49.5 (4.2)	0.31
Body mass index, kg/m^2^	19.8 (1.7)	21.5 (1.2)	0.10
Maximal voluntary contraction and muscle components			
MVC, Nm	76.0 (9.6)	93.8 (22.8)	0.15
Muscle thickness, mm	19 (5)	20 (6)	0.76
Muscle echo intensity, a.u.	73 (3)	50 (6)	<0.01
Pre‐exercise blood pressure			
Systolic blood pressure, mmHg	108 (7)	117 (6)	0.06
Diastolic blood pressure, mmHg	72 (6)	71 (6)	0.69
Mean arterial pressure, mmHg	84 (6)	86 (5)	0.60
Post‐exercise blood pressure			
Systolic blood pressure, mmHg	116 (9)	128 (14)	0.15
Diastolic blood pressure, mmHg	74 (5)	80 (7)	0.18
Mean arterial pressure, mmHg	88 (6)	96 (8)	0.12
Change in (∆)blood pressure from pre‐ to post‐exercise			
∆systolic blood pressure, mmHg	9 (9)	11 (17)	0.75
∆diastolic blood pressure, mmHg	2 (2)	9 (4)	0.01
∆mean arterial pressure, mmHg	4 (4)	10 (8)	0.19

*Note*: Means (standard deviation).

Unpaired *t*‐test were used to compare the groups.

Abbreviations: a.u., arbitrary units; MVC, maximal voluntary contraction; *n*, number of participants.

## 4 DISCUSSION

The major findings of this study were as follows: (Costa et al., 2022) the post‐exercise BP response was greater in older normotensive and SBP‐controlled hypertensive than in SBP‐uncontrolled hypertensive females; (Costa et al., 2020) MVC and muscle thickness were not associated with the change in BP from pre‐ to post‐exercise in older females; (Millar et al., 2009) muscle echo intensity was negatively correlated with the change in DBP from pre‐ to post‐exercise in older normotensive females only. Further, exploratory analysis suggested that the increase in DBP from pre‐ to post‐exercise was smaller in older normotensive females with greater muscle echo intensity than in those with lower echo intensity. These results partly support our hypothesis and suggest that non‐skeletal muscle components, possibly intramuscular fat, affects the post‐exercise BP response in older normotensive but not in SBP‐controlled and ‐uncontrolled hypertensive females.

### 4.1 Different post‐exercise blood pressure response between older normotensive, SBP‐controlled, and ‐uncontrolled hypertensive females

MAP was decreased from pre‐ to post‐exercise in just 1 of 10 normotensives, 2 of 8 SBP‐controlled, but in 8 of 17 hypertensive females. (Figure 1c). Given muscle components such as muscle thickness and echo intensity were similar between normotensive, SBP‐controlled and ‐uncontrolled hypertensive females (Table 1), it seems unlikely the difference between groups in the BP post‐exercise was due to the muscle components.

One of the main mechanisms underlying the post‐exercise reduction in BP is the centrally mediated decrease in sympathetic nerve activity with reduced signal transduction from sympathetic nerve activation into vasoconstriction in normotensive (Halliwill et al., 1996) and hypertensive individuals (Floras & Senn, 1991). Interestingly, Floras and Senn (Floras & Senn, 1991) reported that at rest, muscle sympathetic nerve activity (MSNA) was increased in young borderline hypertensive individuals compared with normotensive individuals. However, BP and MSNA were decreased after exercise in borderline hypertensive but in not normotensive individuals. Although Halliwill et al. (Halliwill et al., 1996) reported that this phenomenon occurred in normotensive individuals as well, it may be that hypertensive individuals, who generally have higher MSNA (Floras & Senn, 1991), exhibit larger transient suppression of augmented central sympathetic outflow post‐exercise than normotensive females.

### 4.2 Association between muscle echo intensity and blood pressure response to post‐exercise in older normotensive females

In the current study, muscle echo intensity (i.e., intramuscular fat) was negatively correlated with ΔDBP, but not ΔSBP or ΔMAP post‐exercise in older normotensive females only. Increased intramuscular pressure during exercise may be associated with this phenomenon. For, Gallagher et al. reported that intramuscular pressure during DBP but not SBP was significantly and continuously elevated during incremental (20 W/min) cycle exercise to fatigue with lower‐limb compression created by using lower body‐positive pressure (Gallagher et al., 2001). This result suggests that intramuscular pressure may be associated with DBP, but not with SBP elevation in response to exercise. Although the association between intramuscular pressure and intramuscular fat remains unknown, a previous study reported that intramuscular pressure was positively (Sadeghi et al., 2019), whereas intramuscular fat was negatively associated with muscle stiffness (Pinel et al., 2021). In this context, it seems reasonable to speculate that a higher muscle echo intensity—an index of greater intramuscular fat—may be associated with lower muscle stiffness, and so result in less of an increase in intramuscular pressure during and/or after exercise. Reduced intramuscular pressure may in turn, contribute to less pressure on the peripheral intramuscular circulation. Future research will be required to clarify the links between intramuscular pressure, intramuscular fat, and muscle stiffness and how they may contribute to differences in the post‐exercise BP response.

### 4.3 Lack of association between muscle echo intensity and blood pressure response at post‐exercise in older blood pressure hypertensive females

Association between BP and muscle echo intensity was not present in the older hypertensive females of the present study. (Figure 5). At least two mechanisms should be considered to account for this lack of association. First, exaggerated sympathetic nerve activity may explain this phenomenon. Narkiewicz et al. reported that females exhibited a greater increase in MSNA with aging, especially when post‐menopausal women were compared with males (Narkiewicz et al., 2005). Further, older hypertensive individuals showed greater MSNA compared with age‐matched normotensive individuals (Yamada et al., 1989). Because MSNA is strongly associated with BP in older but not young females (Narkiewicz et al., 2005), exaggerated MSNA may have a more marked effect on the BP response than the muscle components do, in older hypertensive females.

Second, increased arterial stiffness may contribute to this lack of association. Arterial stiffness increases with aging, particularly, in postmenopausal‐, compared with premenopausal women (Takahashi et al., 2005), and arterial stiffness has emerged as a prominent marker of cardiovascular risk in patients with hypertension (Boutouyrie et al., 2021). Recent research suggests that arterial stiffness mediated the positive association between aging and BP, and that arterial stiffness may precede elevated BP in Asian populations (Wu et al., 2019). Thus, we cannot exclude the possibility that arteries become stiffer, particularly, in older hypertensive females and that this had a greater impact on BP post‐exercise than muscle components in this group.

### 4.4 Comparison with previous studies

In contrast to previous research (Lee, Notay, et al., 2021), absolute MVC was not associated with ∆BP after adjusting for age and BMI in older normotensive, SBP‐controlled or ‐uncontrolled hypertensive females. Loss of muscle strength with aging may have led to this disparity The decrease in muscle strength with aging is due not only to loss of muscle volume (Mitchell et al., 2012) and reduced muscle quality (Pinel et al., 2021), but also to attenuated neural activation properties (Watanabe et al., 2016). Watanabe et al. demonstrated that decreased motor unit firing/recruitment properties were associated with age‐related loss of muscle strength (Watanabe et al., 2016). Attenuation of muscle strength due to age‐related loss of motor unit activation may also result in insufficient muscle strength to affect the BP response during and post‐exercise, in contrast to that previously described in young individuals (Lee, Notay, et al., 2021). Whether loss of muscle strength itself, or some other aspect of muscle strength, such as motor unit activation properties, are associated with the BP response remains unknown.

Differences between the exercise protocols may also explain the lack of association between BP and absolute MVC in the present study. A previous study employed 10 and 30% MVC static (120‐s) and isokinetic dynamic (180‐s; 1:2 work‐to‐rest ratio; angular velocity, 60°s^−1^) knee extensor exercise (Lee, Lutz, et al., 2021). By contrast, in the present study, the exercise was ramp‐up exercise until 50% MVC with 50% MVC static exercise (~30‐s) and five repeated MVCs (10‐s intervals between each contraction). This may have been too small as an exercise stimulus to reveal an association between absolute MVC and the BP response to exercise. It is also important to note that we measured BP immediately after exercise, whereas previous studies measured BP during exercise (Lee, Lutz, et al., 2021; Lee, Notay, et al., 2021; Notay et al., 2018) and in post‐exercise with circulatory occlusion (Lee, Notay, et al., 2021).

### 4.5 Study limitations

The current study had several limitations. First, as noted above, we conducted a very short‐lasting exercise protocol, ramp‐up exercise until 50% MVC with 50% MVC static exercise (~30‐s) and five repeated MVCs (~50‐s). This protocol may be too short and/or too weak to show relationships between MVC and BP reported in a previous study (Lee, Lutz, et al., 2021). However, even with our short‐lasting exercise protocol, we could observe differences between groups for BP post‐exercise. (Figure 2). Second, although the sample size was too small for exploratory analysis of the potential effects of muscle echo intensity on some aspects of post‐exercise BP values in normotensives, we did have sufficient effect size (d, 4.85 and 2.21) and enough power (1‐β, 0.999 and 0.86) to detect a significant difference in our primary outcome—muscle echo intensity and change in DBP from pre‐ to post‐exercise. Nevertheless, we acknowledge that small sample size could have biased our results. Therefore, our findings need to be confirmed in future studies with a larger sample size.

We accept that wrist BP measurement has several limitations of measurement accuracy. In general, SBP increases in more distal arteries, whereas DBP decreases. However, MAP falls by only 1–2 mm Hg between the aorta and peripheral arteries (O'Rourke, 2002). Moreover, we focused on changes in BP relative to pre‐exercise rather than on differences in absolute BP values between groups. It is also the case that wrist BP measurement depends on whether location of wrist is heart level or not (Pickering et al., 2005). Thus, we were very careful to ensure that participants maintained their wrist at heart level during BP measurement.

It is also a limitation that we did not ask participants about their daily physical activity or control the participant's fasting prior to the protocol. Finally, although we have speculated on how sympathetic nerve activity may have affected our results, we did not assess heart rate variability or MSNA to assess the impact of sympathetic neural response. Thus, a future study is warranted to assess the mechanisms underlying the post‐exercise BP response in older normotensive, SBP‐controlled and ‐uncontrolled females.

## 5 CONCLUSION

In conclusion, the findings of our pilot study suggest that intramuscular fat assessed by muscle echo intensity, but not muscle size assessed by muscle thickness is negatively associated with the BP response immediately after exercise in older females. We propose that greater intramuscular fat associates with less muscle stiffness and less intramuscular pressure, resulting in less pressure on the peripheral intramuscular circulation post‐exercise. Further research is needed to clarify the impact of intramuscular fat on BP regulation during and after exercise in a larger cohort of women.

## AUTHORS' CONTRIBUTIONS

Ryosuke Takeda contributed to analysis and interpretation of data; and drafting the article and revising it critically for important intellectual content. Ryosuke Takeda, Tetsuya Hirono, Akito Yoshiko, Shun Kunugi, Masamichi Okudaira, and Saeko Ueda contributed to conception and design of the experiments; collection, analysis, and interpretation of data; and revising the article for important intellectual content. Kohei Watanabe obtained funding support; contributed to collection, analysis, and interpretation of data; and also contributed to drafting the article and revising it critically for important intellectual content. All authors approved the final version of the manuscript. The authors have no disclosures.

## FUNDING INFORMATION

This work was supported by Japan Society for the Promotion of Science (18H03158).

## CONFLICT OF INTEREST

None.
